# Supplementary material for: ProteinShader: illustrative rendering of macromolecules
Source: BMC Struct Biol. 2009 Mar 30;9:19. doi: 10.1186/1472-6807-9-19 (PMC2672931; doi:10.1186/1472-6807-9-19)
Supplement: Additional file 1 — ProteinShader program without source code. This compressed file contains the complete ProteinShader program including associated libraries, but no source code. A README.txt file gives an overview of the ProteinShader distribution, and the index.html file in the help subdirectory has directions on getting started with the program as well as a set of tutorials. [file 1472-6807-9-19-S1.zip › ProteinShader-beta-0_9_4-binary/help/api/allclasses-noframe.html]

All Classes (ProteinShader API)


**All Classes**
  

AAColorEnum
  
AAPortionMode
  
AboutBox
  
AboutBoxListenerFactory
  
AminoAcid
  
AminoAcidEnum
  
AminoAcidLabelVisitor
  
AntialiasingPanel
  
AntialiasingPanelListenerFactory
  
Atom
  
AtomColorPanel
  
AtomEnum
  
AtomModifier
  
AtomScalePanel
  
AtomVisibilityPanel
  
BackgroundMenu
  
BackgroundMenuListenerFactory
  
BetaStrand
  
BetaStrandRecord
  
BetaStrandSegmentFactory
  
Bond
  
BondDestroyerVisitor
  
BondEnum
  
BondGeneratorVisitor
  
BondLengthException
  
BondModifier
  
BondPredictor
  
BoundsVisitor
  
Camera
  
CanvasKeyListener
  
CanvasMouseListener
  
CanvasMouseListener.Mode
  
CartoonColorPanel
  
CartoonSideChainsPanel
  
CartoonSideChainsPanelListenerFactory
  
CartoonVisibilityPanel
  
Category
  
CategoryEnum
  
CategoryEnumDemo
  
CenteredListCellRenderer
  
Chain
  
ColorOutOfRangeException
  
ColorPanel
  
ColorPanelListenerFactory
  
*ConfigReader*
  
ControlPanel
  
CPKColorEnum
  
Cylinder
  
CylinderListInfo
  
CylinderReferences
  
DecorationEnum
  
DecorationsPanel
  
DecorationsPanelListenerFactory
  
Description
  
Drawable
  
DrawableEnum
  
DrawableModifier
  
DrawableSorter
  
EqualSignConfigReader
  
ExtrudedShape
  
FileExtensionFilter
  
FileMenu
  
FileMenuListenerFactory
  
FpsLabel
  
FrenetFrameGeneratorVisitor
  
FrenetFrames
  
GeometricListInfo
  
GLF2GlyphSetFactory
  
GLF2Header
  
Glyph
  
GlyphConfigException
  
GlyphDemo
  
GlyphException
  
GlyphImageException
  
GlyphSet
  
*GlyphSetFactory*
  
GlyphSetFactoryFactory
  
Helix
  
HelixEnum
  
HelixRecord
  
HelixShapeEnum
  
HelpMenu
  
HelpMenuListenerFactory
  
Hermite
  
HermiteDemo
  
Heterogen
  
*IDTest*
  
ImageFileChooser
  
ImageFormatEnum
  
InvalidIDException
  
InvalidRegionException
  
JitterEnum
  
Lighting
  
LocalFrame
  
LocalFrameDemo
  
Loop
  
LoopGeneratorVisitor
  
MainMenuBar
  
*Mediator*
  
MediatorImpl
  
MissingAATypeException
  
MissingAtomTypeException
  
MissingHetNameException
  
Model
  
ModifierPanel
  
ModifierPanelListenerFactory
  
ModifierVisitor
  
ModifierVisitorDemo
  
MotionPanel
  
MotionPanelListenerFactory
  
NamePair
  
OptimizePanel
  
OptimizePanelListenerFactory
  
OrientationEnum
  
OrientationMenu
  
OrientationMenuListenerFactory
  
PaddedListCellRenderer
  
PDBAtomFieldExtractor
  
PDBBetaStrandFieldExtractor
  
PDBConnectFieldExtractor
  
PDBFieldExtractorException
  
PDBFileFilter
  
PDBHelixFieldExtractor
  
PDBLineParser
  
PDBLineParserException
  
PDBStructureReader
  
PDBStructureReaderDemo
  
Point3d
  
ProteinShader
  
ProteinShaderGUI
  
ProteinShaderSplasher
  
Quaternion
  
QuaternionDemo
  
RadioButtonEnum
  
RadioPanel
  
RadioPanel.Type
  
RadioPanelListenerFactory
  
Record
  
RecordEnum
  
RecordEnumDemo
  
Region
  
RegionColorEnum
  
RegionEnum
  
RegionMode
  
Renderer
  
Residue
  
ResidueEnum
  
ResidueMode
  
Ribbon
  
Rotation
  
ScalePanelListenerFactory
  
ScreenShot
  
ScreenShotException
  
Segment
  
SegmentDemo
  
SegmentException
  
SegmentFactory
  
SegmentListInfo
  
SegmentModifier
  
SegmentReferences
  
SegmentRenderer
  
SelectorPanel
  
SelectorPanelListenerFactory
  
SeparatorListCellRenderer
  
SFWriterVisitor
  
ShaderEnum
  
ShaderException
  
ShaderManager
  
ShaderProgram
  
ShaderProgramFactory
  
Shape
  
SideChainEnum
  
Silhouette
  
SlerpDemo
  
Sphere
  
SphereListInfo
  
SphereReferences
  
SpringLoadedJFrame
  
Structure
  
*StructureReader*
  
StructureReaderException
  
StructureToGraphics
  
StyleEnum
  
StyleMenu
  
StyleMenuListenerFactory
  
TextLabel
  
TextLabelFactory
  
TextLabelManager
  
Texture
  
TextureException
  
TextureFactory
  
TextureManager
  
ToolsMenu
  
ToolsMenuListenerFactory
  
Tube
  
TubeVertices
  
Vec3d
  
VectorAndPointDemo
  
VisibilityEnum
  
VisibilityMenu
  
VisibilityMenuListenerFactory
  
VisibilityPanel
  
VisibilityPanelListenerFactory
  
VisibilityVisitor
  
*Visitable*
  
Visitor
  
VisitorDemo
  
VisitorException
  
Water
  
WindowListenerFactory
  
WriterVisitorException
